# Evaluating an evidence-based curriculum in undergraduate palliative care education: piloting a phase II exploratory trial for a complex intervention

**DOI:** 10.1186/1472-6920-13-1

**Published:** 2013-01-04

**Authors:** Christian Schulz, Mischa F Möller, Daniel Seidler, Martin W Schnell

**Affiliations:** 1 University Dusseldorf, Medical Faculty, Interdisciplinary Centre for Palliative Medicine, Moorenstraße 5, D-40225, Dusseldorf, Germany; 2 Institute for Ethics and Communication in Healthcare Systems, School of Medicine, Faculty of Health, Witten/Herdecke University, Alfred-Herrhausen-Straße 50, D-58448, Witten, Germany; 3 University Dusseldorf, Medical Faculty, Clinical Institute for Psychosomatic Medicine and Psychotherapy, Moorenstraße 5, D-40225, Dusseldorf, Germany

## Abstract

**Background:**

By 2013 Palliative Care will become a mandatory examination subject in the medical curriculum in Germany. There is a pressing need for effective and well-designed curricula and assessment methods. Debates are on going as how Undergraduate Palliative Care Education (UPCE) should be taught and how knowledge and skills should be assessed. It is evident by this time that the development process of early curricula in the US and UK has led to a plethora of diverse curricula which seem to be partly ineffective in improving the care for the seriously ill and dying offered by newly qualified doctors, as is demonstrated in controlled evaluations. The goals of this study were to demonstrate an evidence-based approach towards developing UPCE curricula and investigate the change in medical students’ self-perceived readiness to deal with palliative care patients and their families.

**Methods:**

To evaluate the effects of the UPCE curriculum we chose a prospective, controlled, quasi-experimental, pre, retrospective-pre, post study design. A total of n = 37 3^rd^ and 4^th^ –year medical students were assigned to the intervention group (n = 15; 4^th^ -year) and to the control group (n = 22; 3^rd^-year). Resting on the self-efficacy concept of Bandura the measurement was conducted by a refined test-battery based on two independent measurements (the revised Collet-Lester-Fear-of-Death-Scale and the instrument of the “Program in Palliative Care Education and Practice” at Harvard Medical School) including 68 items altogether in a five-point Likert-scale. These items were designed to test elementary skills in caring for the dying and their relatives as perceived by medical undergraduates. Datasets from both groups were analysed by paired and independent two-sample t-test. The TREND statement for reporting non-randomized evaluations was applied for reporting on this quasi-experimental study.

**Results:**

Three constructs showed statistically significant differences comparing the intervention group before and after. Willingness to accompany a dying patient increased from 21.40 to 37.30 (*p* < .001). Self-estimation of competence in communication with dying patients and their relatives increased from 12.00 to 23.60 (*p* = .001). Finally, self-estimation of knowledge and skills in Palliative Care increased from 8.30 to 13.20 (*p* = .001).

**Conclusions:**

This study is a small but systematic step towards rigorous curricular development in palliative care. Our manualised curriculum is available for scrutiny and scientific feedback to support an open and constructive process of best-practice comparison in palliative care.

## Background

The need for Palliative Care (PC) was acknowledged by the European Council at the beginning of the century [1]. The task had been set up to establish PC systematically in the European Union. Later, the European Parliament, in order to build-up PC in healthcare-systems, emphasized the specific training needs of professionals and the demand for systematic training programmes [2]. In 2009, supported by a broad civil movement, the German legislation passed a law that introduced an obligation to teach undergraduate palliative care education (UPCE) within the medical curriculum. German universities were obliged to develop and implement curricula by 2013. A German survey conducted at the same time showed that by then only six out of 36 medical schools offered mandatory UPCE, but most faculties offered some kind of teaching in PC [3]. Although there are recommendations available from the European Association of Palliative Care and national organisations for curriculum planning, debates are ongoing as to how UPCE should be taught and how knowledge and skills should be assessed [4]. In comparison the movement to improve UPCE in medical schools in the US and UK started earlier in the late 1980s resulting in the implementation of UPCE, e.g. in the UK in 1993 [5,6]. It is evident by this time that the development process of those early curricula has led to a plethora of diverse curricula which, most importantly, seem to be partly ineffective in improving the care for the seriously ill and dying offered by newly qualified doctors, as demonstrated in controlled evaluations [7-9]. In 2004 a systematic review found a lack of consistency in analysed UPCE curricula focussing on knowledge and skills teaching rather than on attitude. Furthermore they recognised rare formal assessment [10]. This has been confirmed by a more recent systematic review of the US situation [11]. We do see parallels between the implementation process of UPCE in the US/UK and today’s situation in Germany where recent findings demonstrate that final year medical students show only limited confidence and knowledge towards palliative care [12]. Therefore we hypothesise that the efforts taken in developing UPCE curricula in Germany need to be strongly supported by medical education expertise and evidence-based approaches.

This report is part of a series of systematic investigations demonstrating an evidence-based approach towards developing UPCE curricula [13]. The goal of this study was to investigate the change in medical students’ self-perceived readiness to deal with palliative care patients and their families.

## Methods

### Participants

The study was conducted at the University Witten/Herdecke as a new part of the medical curriculum from September 2006 to April 2007. Ethical approval was obtained from the Institutional Review Board of the university (*“Ethik-Kommission der Universität Witten/Herdecke”*). With regards to the naturalistic design of this study and a small cohort of 4^th^ -year medical students we had to recruit 3^rd^ -year medical students as controls. Randomisation was impossible for pragmatic and ethical reasons. Recruitment was performed via email-invitation to the registered addresses at the registrar’s office in September 2006 to all 3^rd^ and 4^th^ -year medical students. Written consent was obtained from all participants (n = 37). An overview in participant flow is given in Table 1. Exposure to the intervention took place at the facilities of the university between October 2006 and February 2007. Data-collection was performed via a web portal [14] before (T1) and immediately after the intervention (T2).

**Table 1:** Participant flow

	**Target population**	**T1 (participation)**	**T2 (response rate)**	**Dropouts (percentage)**
Intervention group	43	15 (.35)	10 (.67)	5 (.33)
Control group	44	22 (.50)	16 (.73)	6 (.27)
Total	87	37 (.43)	26 (.70)	11 (.30)

This table shows the flow of participating students during the study time-points T1 and T2. Response rates and dropout rates were calculated for the study cohort at T1. Dropouts resulted from lost contact.

Eligibility criteria were: (1) medical undergraduates at Witten/Herdecke, (2) students have to be in their 3^rd^ or 4^th^ year of study and (3) students in the intervention group (IG) must agree to complete the whole intervention. Two incentive study credits were rewarded to participants in the IG. No incentives were offered to the control group (CG) whose participants were confronted with considerably less workload during participation. In general, the new UPCE curriculum was accessible for all students, including those who did not consent to participate in the evaluation study. To assure psychological support during potential stressful confrontations with palliative patients one psychologist trained in psycho-oncology was introduced prior to the commencement of the intervention and offered immediate help to the participants by phone during the whole study.

### Intervention

**Figure 1:**
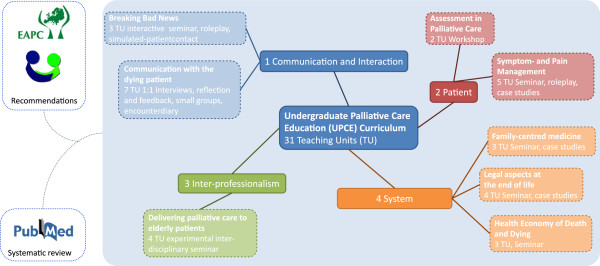
**UCPE curriculum.** Based on a systematic review of literature, four central dimensions of PC emerged: (1) Communication and Interaction, (2) Patient assessment and management, (3) Inter-professionalism and (4) Systemic aspects.

The manualised UPCE curriculum was used as an intervention in this study. The Institute for Healthcare Ethics and Communication (IEKG) introduced the curriculum first in 2006 at Witten/Herdecke University. Based on a systematic review of literature we developed the curriculum according to Kern’s approach to curriculum development, a six-step framework for evidence-based curricular development [15]. The underlying literature review cannot be fully reported in this article. Four contextual teaching domains evolved during our synthesis summarised in Figure 1: (1) communication and interaction, (2) patient assessment and management, (3) inter-professionalism and (4) systemic aspects. The curriculum consists of a total of 31 teaching units (TU = 45mins) taught to fourth-year medical students during the course of 2 semesters. According to recommendations for teaching communication in medicine, preferred didactic methods should be interactive, focussing on group discussion, teamwork, role-play and patient exposure [16]. A precise manual of the developed UPCE curriculum and the respective teaching units can be found as Additional file 1 in the online section for this article. This publication assesses the effect on medical students completing the whole UPCE curriculum as an intervention, while a series of publications covers detailed analyses of the curriculum’s modules [17,18]. Qualitative analysis of encounters between dying patients and medical students during real patient contact in this curriculum has been covered by a master thesis at King’s College London, UK.

### Objectives

**Figure 2:**
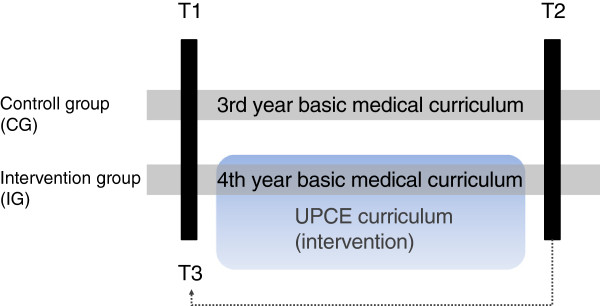
**Study design.** T1 and T2 represent the record of the pre and post dataset.

To evaluate the effects of the UPCE curriculum we chose a prospective, controlled, quasi-experimental, pre, retrospective-pre, post study design. The TREND statement for reporting non-randomized evaluations was applied for reporting on this quasi-experimental study [19]. Due to the small number of participants this study needs to be considered as a pilot trial. The primary outcome was to measure self-efficacy in several aspects of PC in medical undergraduates. We investigated two cohorts of students: the IG consisted of 4^th^ -year students, who passed the UPCE curriculum. 3^rd^ –year students were assigned to the CG passing the standard medical curriculum. Both groups were tested at T1 and T2 using our measurements described below. T2 assesses post-intervention status, while T3 represents the retrospective-pre estimates in time-point T1 (overview in Figure 2). The retrospective-pre study design was used to assess a potential response-shift. It is known from previous medical and psychology research that respondents overrate their knowledge or skills prior to training [20]. Therefore interventions that improve skills or knowledge more effectively should demonstrate a higher difference comparing pre and retrospective-pre data of estimates in self-efficacy. This approach has been used successfully in a similar setting in Palliative Care Education research [21].

### Outcomes

To investigate the effect of the UPCE curriculum on the student cohort we used the self-efficacy concept with controls to minimize potential confounders [22]. This concept is well-known and has already been used in UPCE research [23-29]. We created a refined test-battery based on two independent measurements. Firstly, we used 36 items of a published instrument from the “Program in Palliative Care Education and Practice” (PCEP) at Harvard Medical School to obtain information about self-estimation in knowledge, skills and attitude towards PC [30]. Secondly, we administered the revised Collet-Lester-Fear-of-Death-Scale [31], a 32 items instrument on attitudes towards death and dying of self and others, which had been used in palliative care education research before [32]. The rationale for the addition of this second instrument was the existing evidence from death education research, which shows that death education programs with an experiential focus can lead to a change in death-related cognitions [33] and can also achieve a modification in death-related behaviours [34]. The combined questionnaire consisted of 68 items and a five-point Likert-scale design with semantic differentials. This 68-item instrument was translated into German followed by a validation in form of a two-step-Delphi process [35]. Seven experts in palliative medicine and palliative care education were contacted with a response rate of 100% for both steps. The Delphi discussion was enabled through an online platform that allowed for anonymous commentaries and feedback from each expert for each single item of the instrument. As a result, the Delphi process led to semantic changes in three of the translated items (data not shown, details are readily available upon request). Afterwards, a pre-test was performed to control comprehensibility and internal consistency. We measured a total Cronbach’s Alpha of .91 (Table 2). As independent variables we assessed five constructs essential for PC: (1) attitude towards PC, (2) willingness to accompany a dying patient, (3) self-estimation of competence in communication with dying patients and their relatives, (4) self-estimation of knowledge and skills in providing PC and finally (5) attitude towards death and dying of self and others. The last construct was divided into four subsections to investigate (5.1) attitude towards death of self, (5.2) attitude towards dying of self, (5.3) attitude towards death of others and finally (5.4) attitude towards dying of others. Additional demographic characteristics like age, pre-education in PC, medical interest, contact to a dying patient during medical school and experiences of personal bereavement were assessed.

**Table 2:** Internal consistency

**Construct**	**Items**	**Cronbach’s Alpha**
(1) Attitude towards palliative care	12	.28
(2) Willingness to accompany dying patients	13	.91
(3) Self- estimation of competence in communication with dying patients and their relatives; (4) self-estimation of knowledge and skills in PC	11	.85
(5.1) (5.2) Attitude towards death and dying of self	16	.90
(5.3) (5.4) Attitude towards death and dying of others	16	.83
Total	68	.91

The first dimension did not meet the criteria for reliability and was excluded from data analysis.

### Sample size

Unfortunately, the maximum study size was not modifiable due to the naturalistic design of the study, which took place in a medical curriculum with pre-existing student cohorts.

### Assignment method

Randomisation or matching was not possible due to the naturalistic design of the study. 4^th^ -year medical students were assigned to the IG to graduate the new UPCE curriculum, while 3^rd^ -year students were assigned to the CG only, passing the standard medical curriculum. We could not exclude selection bias because of the missing randomisation. One can hypothesize that participants with a higher interest in PC were more likely to participate than unmotivated students (in both groups).

### Blinding

There was no possibility of blinding in this setting.

### Unit of analysis

Analyses were performed at the individual level of each student participant.

### Statistical methods

The data from the new instrument was collected anonymously via a password-secured web portal from the IG and CG each at T1 and T2/T3 (http://www.palliative-research.de). Datasets from both groups at T1, T2 and T3 were processed with SPSS® 19.0 and analysed by dependent t-test for paired samples (e.g. T1 IG vs. T2 IG) and independent two-sample t-test (e.g. T1 IG vs. T1 CG). Levene’s test was applied to prove equality of variances. *P-value* for two-tailed statistical significance was predefined to *p <* .05. Only retrospective-pre data of previously significant constructs in pre-post comparison was tested.

## Results

### Participant flow

A total of 87 students met the inclusion criteria and were invited to participate in the study. In T1 n = 37 students completed the questionnaire (IG n = 15; CG n = 22). In T2 n = 26 students remained (IG n = 10; CG n = 16). Total response rate in T1 was 0.43 (IG 0.35; CG 0.50) compared to the total response rate in T2 of 0.30 (IG 0.23; CG 0.36). We had n = 11 dropouts representing a dropout rate of 0.30 in total (IC 0.33; CG 0.27). We did not find a difference in compliance between IG and CG (Fisher’s exact test .73). Six dropouts resulted from lost contact and five students had to abort the curriculum due to electives in foreign countries. An overview of participant flow can be seen in Table 1.

### Recruitment

Recruitment started in September 2006 and ended in October with T1 and the beginning of the UPCE curriculum. Follow-up in T2 was performed in February 2007 at the end of the intervention.

### Baseline data

Participants in both groups had no prior formal education in PC. The mean age in both groups was 24 years (standard deviation [SD]: IG 2.971; CG 2.428). Notably 84% of participants had contact to dying patients during medical school without significant differences between the groups. Most of those contacts took place in hospital in-patient settings. More details can be found in Table 3 and in Additional file 2.

**Table 3:** Contact with dying patients before trial participation

	**IG (n = 15)**	**CG (n = 22)**	**total (n = 37)**	***p***
yes	14	17	31	.193
No	1	5	6	
	(n = 14)	(n = 17)	(n = 31)	
Setting:				
Standard care	10	16	26	.087
Palliative care	4	1	5	.087
Hospice	0	2	2	.185
Nursing home	1	4	5	.217
Ambulant care	3	8	11	.138

Participating students were asked if they had had contact to a dying patient before T1 and in which setting the encounter happened.

### Baseline equivalence

None of the measured demographic parameters of the participants in the IG or the CG differed at a statistically significant level (p < .05). Pre-intervention comparison of both groups (T1; IG vs. CG) showed slight but significant differences in two subsections of the revised Collet Lester scale: (5.2) attitude towards dying of self. The mean value of the IG was 12.33 (SD 2.41) compared to 15.23 (SD 5.07) in the CG (*p =* .027). Levene’s test showed inequality of variances (F = 6.67; *p* = .01). In (5.3) attitude towards death of others mean values were 15.13 (SD 3.46) in the IG and 17.95 (SD 4.04) in the CG (*p =* .6). Levene’s test proved equality of variances (F = .28; *p* = .04). Results are summarised in Table 4.

**Table 4:** Baseline intergroup-comparison at T1

**Construct (No.)**	**Groups**	**T1 M ± SD**	***t***	***Df***	***p*-value**	**MD**	**95%CI**
(2) Willingness to accompany dying patients	IG	24.7 ± 7.17	.07	35	.948	.16	−4.72, 5.03
	CG	23.91 ± 7.17					
(3) competence in communication with dying patients and their relatives	IG	14.53 ± 5.4	.56	35	.582	.94	−2.5, 4.38
	CG	13.59 ± 4.83					
(4) self-estimation of knowledge and skills in PC	IG	8.27 ± 1.87	1.11	35	.276	.72	-.6, 2.04
	CG	7.55 ± 1.99					
(5.1) Attitude towards death of self	IG	22.2 ± 8.71	.79	35	.433	−2.03	−7.21, 3.16
	CG	24.2 ±6.82					
(5.2) Attitude towards dying of self	IG	12.33 ± 2.41	−2.32	31.96	.027 *	−2.89	−5.43, -.35
	CG	15.23 ± 5.07					
(5.3) Attitude towards death of others	IG	15.13 ± 3.46	−2.19	34	.036 *	−2.82	−5.44, -.2
	CG	17.95 ± 4.04					
(5.4) Attitude towards dying of others	IG	18.6 ± 5.36	-.25	34	.808	-.4	−3.72, 2.92
	CG	19.0 ± 4.43					

Legend: (2) Willingness to accompany dying patients; (3) Self-estimation of competence in communication with dying patients and their relatives; (4) self-estimation of knowledge and skills in PC; (5.1) Attitude towards death of self; (5.2) Attitude towards dying of self; (5.3) Attitude towards death of others; (5.4) Attitude towards dying of others. M = mean; SD = standard deviation; MD = mean difference; * significant.

Baseline comparison shows equality of both groups with exception of construct (5.2) and (5.3). Data of (1) is not presented because that construct has proved to be unreliable.

### Numbers analysed

In T1 data was obtained from n = 37 participants (IG n = 15; CG n = 22). In T2 the number of data sets was n = 26 (IG n = 10; CG n = 16). We had to note n = 11 dropouts, which were excluded from analysis.

### Outcomes and estimation

From the five constructs under investigation three showed statistically significant differences comparing the IG pre and post: (2) willingness to accompany a dying patient, (3) self-estimation of competence in communication with dying patients and their relatives and (4) self-estimation of knowledge and skills in PC. In (2) the mean value increased from 21.40 to 37.30 (SD 6.82/6.65; *p* < .001). The mean value in (3) increased from 12.00 to 23.60 (SD 4.32/5.58; *p* = .001). Results of (4) showed an increase in the mean value from 8.30 to 13.20 (SD 2.11/2.70; *p* = .001). To get an overview of the results and for additional data of non- significant constructs see Table 5. What is more, there were no statistical significant differences for the CG in T1 and T2. Post-interventional comparison of IG and CG at T2 confirmed these findings. The data shows statistical significant results in favour of the IG for constructs (2), (3) and (4) in accordance with the previous results in Table 5 (overview in Additional file 3). Surprisingly, the retrospective-pre analysis did not reveal a response-shift in the IG between T1 vs. T3 (data not shown).

**Table 5:** Intragroup-comparison between T1 and T2

**Construct (No.)**	**Groups**	**T1 M ± SD**	**T2 M ± SD**	***t***	***df***	***p*-value**	**MD**	**95%CI**
(2)	IG	21.4 ± 6.82	37.3 ± 6.65	−9.2	9	<.001 *	−15.9	−19.81, -11.99
	CG	22.56 ± 7.05	24.19 ± 7.53	-.7.9	15	.44	−1.63	−6.01, 2.76
(3)	IG	12.0 ± 4.32	23.6 ± 5.58	−4.8	9	.001 *	−11.6	−17.07, -6.13
	CG	14 ± 5.02	13.69 ± 6.12	.22	15	.83	.31	−2.68, 3.31
(4)	IG	8.3 ± 2.11	13.2 ± 2.7	−4.62	9	.001 *	−4.9	−7.3, -2.5
	CG	7.88 ± 2.19	8.75 ± 2.89	−1.73	15	.11	-.88	−1.96, .21
(5.1)	IG	18.8 ± 5.43	20.0 ± 4.19	-.73	9	.48	−1.2	−4.91, 2.51
	CG	23.88 ± 6.52	23.75 ± 6.28	.1	15	.92	.13	−2.56, 2.81
(5.2)	IG	11.3 ± 2.0	14 ± 4.32	−1.52	9	.163	−2.7	−6.72, 1.32
	CG	14.94 ± 5.18	15.63 ± 6.12	-.77	15	.45	-.69	−2.59, 1.22
(5.3)	IG	13.6 ± 2.95	15.9 ± 3.96	−1.8	9	.11	−2.3	−5.18, .58
	CG	18.06 ± 4.45	17.88 ± 5.11	.22	15	.83	.19	−1.63, 2.0
(5.4)	IG	17.7 ± 6.07	21.8 ± 7.01	−1.76	9	.112	−4.1	−9.36, 1.16
	CG	19.25 ± 4.82	20.25 ± 5.64	−1.09	15	.293	−1.0	−2.96, .96

Legend: (2) Willingness to accompany dying patients; (3) Self-estimation of competence in communication with dying patients and their relatives; (4) self-estimation of knowledge and skills in PC; (5.1) Attitude towards death of self; (5.2) Attitude towards dying of self; (5.3) Attitude towards death of others; (5.4) Attitude towards dying of others. M = mean; SD = standard deviation; MD = mean difference; * significant.

Comparison in the groups between T1 and T2 shows statistical significant results for constructs (2), (3) and (4). Data of (1) is not presented because that construct has proved to be unreliable.

### Ancillary analyses

We measured changes in emotional involvement of the participants while attending the seminar “Communication with the dying patient” which fosters death-awareness. Therefore the IG was assessed by a modified version of Izard’s Differential Emotions Scale (mDES) pre and post [36]. The data is provided in Additional file 4.

### Adverse events

There was no need for psychological intervention as reported by the accompanying psychologist. The participants did not demand for psychological help throughout the study.

## Discussion

### Interpretation

The presented pilot study is the first one to measure and report effects of a manualised and systematically implemented UPCE curriculum in Germany. Systematic implementation and detailed evaluation are most important for high quality medical education in palliative care. The quasi-experimental design of this study does not allow proofing for causal relations. However, our statistical results can inform further research by demonstrating associations, e.g. between an evidence-based curriculum in UPCE adopting modern teaching methods and a rise in self-efficacy in elementary skills in caring for the dying and their relatives as perceived by medical undergraduates. The UPCE curriculum seems to have the potential to foster self-efficacy in the following domains: willingness to accompany a dying patient, competence in communication with dying patients and their relatives, and knowledge and skills in PC. The self-efficacy concept was chosen for several reasons: firstly, this concept allows testing for a central paradigm in palliative care which values the importance of patient-physician-relationship depending on personal qualities of engagement, self-confidence, self-reflection and patient-centred perspective. Secondly, this concept has already been used in various settings investigating the effects of UPCE [24-31]. And thirdly, from a research ethics point of view this model was economical to use and its measurement easy to apply.

In our ancillary analyses we found hints that palliative care education may have an emotional impact on medical students. Furthermore, these results indicate that those emotional states, which block effective learning, particularly shame, decreased during a seminar about “Communication with the dying patient”.

Unfortunately, some sources of bias could not be avoided in this approach. Randomisation was not possible because of the small study-population and therefore we have to expect self-selection bias. The use of statistical tests on a small sample implies it’s own risks to overestimate the results as well. In this context we can only speak of “association”, not “effect”. Furthermore, self-selection could have brought more motivated students into the IG, leading to over-estimation of the effect-size in the results. Such critical appraisal aspects need to be taken in consideration when applying the study design to a larger sample of medical students.

### Generalisability

This UPCE curriculum consists of 31 teaching units with different didactic methods and approaches. It therefore forms a complex intervention. According to the Medical Research Council framework for complex interventions, research at a high level of complexity should follow a step-wise approach [37]. To gain a systematic insight into this UPCE curriculum we chose an exploratory phase (II) trial design following the MRC recommendations. Several limitations applied to this trial. The study used the implementing of a new curriculum as an intervention (quasi-experimental) and therefore we had to accept the low target population and the resulting low number of participants, which was limited by the given number of eligible students. Calculation of statistical power was not possible in this scenario, reducing the generalisation of the results. We offered incentives only in the IG but surprisingly participation was higher in the CG (.35% vs. 50%), which hints at the perceived workload that medical students might have associated with undergoing this curriculum. This is further strengthened by the high dropout-rate in the intervention group. This highlights the question of mandatory undergraduate training in palliative care, an ongoing debate in Germany [38]. We did measure some baseline differences in one construct concerning fear of death but this did not have any recognizable effect on the interpretation of the results in the other constructs.

### Overall evidence

Complex interventions such as the UPCE curriculum pose particular problems for evaluation trials and this has led to a debate as to which method should be used. On the one hand, randomised controlled trials are widely accepted as the most reliable (“gold standard”) method of determining effectiveness [39,40]. On the other hand, the role of observational methods [41] and the value of qualitative research [42] in evaluation of palliative care services has been underlined by others. Consequently, methodological frameworks have been developed to integrate different research paradigms into a sound research strategy [37]. Being conscious of the lacking possibility of generalisation from a phase (II) study, we do recommend conducting phase (III) or (IV) trials with higher study populations and a more robust design to provide more precise study results, and to encourage research in this field. However, we believe we are giving an example of a step-wise and systematic approach towards palliative care education development and evaluation. In a period of time where constituting PC in medical faculties in Germany is still ongoing, we want to emphasize the importance of a systematic approach towards curriculum development, implementation and evaluation following established principles from medical education expertise. In Germany, promising initiatives have begun to form, which are intended to connect and interlink curricular developers and leading experts in palliative care education [43].

## Conclusions

By 2013 Palliative Care will become a mandatory examination subject in the medical curriculum in Germany. There is a pressing need for effective and well-designed curricula and assessment methods. It would be wise to learn from international experience and take established didactic evidence into account. Development, implementation and evaluation of evidence-based curricula should be fostered by drawing expertise from medical education specialists and intense interfaculty collaboration. This study is a small but systematic step towards rigorous curricular development in palliative care. By making our manualised curriculum readily available for scrutiny and scientific feedback we want to support an open and constructive process of best-practice comparison and high-quality education in palliative care.

## Abbreviations

IG: Intervention group; CG: Control group; PC: Palliative care; PCEP: Palliative care education and practice; SD: Standard deviation; UPCE: Undergraduate palliative care education.

## Competing interests

The authors declare that they have no competing interests.

## Authors’ contributions

CS developed the curriculum initially and was responsible for patient contacts and the manuscript. MFM organized the curriculum, took care of the participating students and was responsible for the manuscript. DS performed the statistical analyses and revised the manuscript. MWS was responsible for the implementation of the curriculum in the medical undergraduate curriculum at Witten/Herdecke University. He did the communication with important gate-keeping contacts and revised the manuscript. All authors read and approved the final manuscript.

## Pre-publication history

The pre-publication history for this paper can be accessed here:

http://www.biomedcentral.com/1472-6920/13/1/prepub

## Supplementary Material

Additional file 1 **Manual_UPCE_curriculum_Witten_2012.pdf.** A manualised model curriculum in Undergraduate Palliative Care Education at Witten/Herdecke University. PDF-viewer required. Click here for file

Additional file 2 **Medical_interest_of_participants.pdf.** Prior to the intervention the medical interest of participants was evaluated. PDF-viewer required. Click here for file

Additional file 3 **Post-interventional_intergroup-comparison_T2.pdf.** Surplus statistical results, confirming the main results. PDF-viewer required. Click here for file

Additional file 4 **Changes_in_emotional_involvement.pdf.** Emotional changes measured by the modified Differential Emotions Scale of the participants while attending the seminar “Communication with the dying patient”. PDF-viewer required. Click here for file

## References

[B1] Assembly P Protection of the human rights and dignity of the terminally ill and the dying Recommendation 1418 (1999) 1999 European Union: Official Gazette of the Council of Europe

[B2] Martin-Moreno J Harris M Gorgojo L Clark D Normand C Centeno C Palliative Care in the European Union 2008 European Parliament: Policy Department EaSP

[B3] Laske A Dietz I Ilse B Nauck F Elsner F Palliativmedizinische Lehre in Deutschland: Bestandsaufnahme an den medizinischen Fakultäten 2009 Z Palliativmed 2010 11 18 25 10.1055/s-0029-1223482

[B4] Elsner F Fittkau-Tönnesmann B Schiessel C Curriculum: Grundlagen der Palliativmedizin 2009 Berlin: Deutsche Gesellschaft für Palliativmedizin

[B5] Field D Wee B Preparation for palliative care: teaching about death, dying and bereavement in UK medical schools 2000–2001 Med Educ 2002 36 6 561 567 10.1046/j.1365-2923.2002.01232.x 12047672

[B6] Council GM Tomorrow's Doctors: Recommendations on Undergraduate Medical Education 1993 London: GMC

[B7] The SUPPORT Principal Investigators A controlled trial to improve care for seriously ill hospitalized patients. The study to understand prognoses and preferences for outcomes and risks of treatments (SUPPORT) JAMA 1995 274 20 1591 1598 10.1001/jama.1995.03530200027032 7474243

[B8] Oddi LF Cassidy VR The message of SUPPORT: study to understand prognosis andpreferences for outcomes and risks of treatment. Change is long overdue J Prof Nurs 1998 14 3 165 174 10.1016/S8755-7223(98)80092-6 9610025

[B9] Gibbins J McCoubrie RaF K Why are newly qualified doctors unprepared to care for patients at the end of life? Medical Education 2011 45 389 399 10.1111/j.1365-2923.2010.03873.x 21401687

[B10] Lloyd-Williams M MacLeod RD A systematic review of teaching and learning in palliative care within the medical undergraduate curriculum Med Teach 2004 26 8 683 690 10.1080/01421590400019575 15763870

[B11] Bickel-Swenson D End-of-life training in U.S. medical schools: a systematic literature review J Palliat Med 2007 10 1 229 235 10.1089/jpm.2006.0102.R1 17298271

[B12] Weber M Schmiedel S Nauck F Alt-Epping B Knowledge and attitude of final - year medical students in Germany towards palliative care - an interinstitutional questionnaire-based study BMC Palliat Care 2011 10 19 10.1186/1472-684X-10-19 22112146 PMC3235960

[B13] Just JM Schulz C Bongartz M Schnell MW Palliative care for the elderly-developing a curriculum for nursing and medical students BMC Geriatr 2010 10 66 10.1186/1471-2318-10-66 20854665 PMC2955033

[B14] Palliative Research Palliative Researchhttp://www.palliative-research.de

[B15] Kern DE Thomas PA Howard DA Curriculum Development for MedicalEducation - A Six-Step Approach 1998 Baltimore, Maryland: John Hopkins University Press http://www.amazon.com/Curriculum-Development-Medical-Education-Six-Step/dp/0801893674

[B16] Moher D Liberati A Tetzlaff J Altman DG Preferred reporting items for systematic reviews and meta-analyses: the PRISMA statement BMJ 2009 339 b2535 10.1136/bmj.b2535 19622551 PMC2714657

[B17] Just JM Schnell MW Bongartz M Schulz C Exploring effects of interprofessional education on undergraduate students behaviour: a randomized controlled trial Journal of Research in Interprofessional Practice and Education 2010 1 3 182 199

[B18] Schulz C Möller MF Schmincke-Blau I Schnell MW Nauck F Communication with the dying patient – Results of a controlled intervention study on communication skills in undergraduates European Journal of Palliative Care. Volume 11 2009 Vienna: Hayward Medical Communications 152

[B19] Des Jarlais DC Lyles C Crepaz N Improving the reporting quality of nonrandomized evaluations of behavioral and public health interventions: the TREND statement Am J Public Health 2004 94 3 361 366 10.2105/AJPH.94.3.361 14998794 PMC1448256

[B20] Pratt CC McGuigan WM Katzev AR Measuring program outcomes: using retrospective pretest methodology Am J Eval 2000 21 3 341 349

[B21] Sullivan AM Lakoma MD Billings JA Peters AS Block SD Teaching and learning end-of-life care: evaluation of a faculty development program in palliative care Acad Med 2005 80 7 657 668 10.1097/00001888-200507000-00008 15980082

[B22] Bandura A Self-efficacy: toward a unifying theory of behavioral change Psychol Rev 1977 84 2 191 215 847061 10.1037//0033-295x.84.2.191

[B23] Adriaansen MJ van Achterberg T A test instrument for palliative care Int J Nurs Stud 2004 41 1 107 117 10.1016/S0020-7489(03)00073-7 14670400

[B24] Fineberg IC Wenger NS Forrow L Interdisciplinary education: evaluation of a palliative care training intervention for pre-professionals Acad Med 2004 79 8 769 776 10.1097/00001888-200408000-00012 15277134

[B25] Fraser HC Kutner JS Pfeifer MP Senior medical students' perceptions of the adequacy of education on end-of-life issues J Palliat Med 2001 4 3 337 343 10.1089/109662101753123959 11596545

[B26] Kaye JM Loscalzo G Learning to care for dying patients: a controlled longitudinal study of a death education course J Cancer Educ 1998 13 1 52 57 9565863 10.1080/08858199809528513

[B27] Mason S Ellershaw J Assessing undergraduate palliative care education: validity and reliability of two scales examining perceived efficacy and outcome expectancies in palliative care Med Educ 2004 38 10 1103 1110 10.1111/j.1365-2929.2004.01960.x 15461656

[B28] Ross DD Shpritz D Hull MM Goloubeva O Long-term evaluation of required coursework in palliative and end-of-life care for medical students J Palliat Med 2005 8 5 962 974 10.1089/jpm.2005.8.962 16238509

[B29] Schwartz CE Clive DM Mazor KM Ma Y Reed G Clay M Detecting attitudinal changes about death and dying as a result of end-of-life care curricula for medical undergraduates J Palliat Med 2005 8 5 975 986 10.1089/jpm.2005.8.975 16238510

[B30] Sullivan AM Lakoma MD Block SD The status of medical education in end-of-life care: a national report J Gen Intern Med 2003 18 9 685 695 10.1046/j.1525-1497.2003.21215.x 12950476 PMC1494921

[B31] Lester D The factorial structure of the revised Collett-Lester fear of death scale Death Stud 2004 28 8 795 798 10.1080/07481180490483472 15446287

[B32] Hegedus K Zana A Szabo G Effect of end of life education on medical students' and health care workers' death attitude Palliat Med 2008 22 3 264 269 10.1177/0269216307086520 18477721

[B33] Durlak JA Reisenberg LA The impact of death education Death Stud 1991 15 1 39 58 10.1080/07481189108252408

[B34] Neimeyer RA Death Anxiety Handbook: Research, Instrumentation, and Application 1994 London: Taylor & Francis

[B35] Hasson F Keeney S McKenna H Research guidelines for the Delphi survey technique J Adv Nurs 2000 32 4 1008 1015 11095242

[B36] Boyle GJ Reliability and validity of Izard's differential emotions scale Pers Indiv Differ 1984 5 6 747 750 10.1016/0191-8869(84)90124-7

[B37] Campbell M Fitzpatrick R Haines A Kinmonth AL Sandercock P Spiegelhalter D Tyrer P Framework for design and evaluation of complex interventions to improve health BMJ 2000 321 7262 694 696 10.1136/bmj.321.7262.694 10987780 PMC1118564

[B38] Weber M Schmiedel S Nauck F Alt-Epping B Knowledge and attitude of final - year medical students in Germany towards palliative care - an interinstitutional questionnaire-based study BMC Palliat Care 2011 10 19 http://www.biomedcentral.com/1472-684X/10/19 10.1186/1472-684X-10-19 22112146 PMC3235960

[B39] Stephenson J Imrie J Why do we need randomised controlled trials to assess behavioural interventions? BMJ 1998 316 7131 611 613 10.1136/bmj.316.7131.611 9518919 PMC1112639

[B40] Simon S Higginson IJ Evaluation of hospital palliative care teams: strengths and weaknesses of the before-after study design and strategies to improve it Palliat Med 2009 23 1 23 28 18952748 10.1177/0269216308098802

[B41] Black N Why we need observational studies to evaluate the effectiveness of health care BMJ 1996 312 7040 1215 1218 10.1136/bmj.312.7040.1215 8634569 PMC2350940

[B42] Flemming K Adamson J Atkin K Improving the effectiveness of interventions in palliative care: the potential role of qualitative research in enhancing evidence from randomized controlled trials Palliat Med 2008 22 2 123 131 10.1177/0269216307087319 18372377

[B43] QB 13 - Palliativmedizin als Pflichtfach im Medizinstudium - Rückblick auf den 2. Dozentenworkshop http://www.cio-koeln-bonn.de/mediziner/neuigkeiten-aktuelles/news-details/detail/qb-13-palliativmedizin-als-pflichtfach-im-medizinstudium-rueckblick-auf-den-2-dozentenworkshop/?tx_ttnews[backPid]=147&cHash=aa76a4a2f9B

