# Supplementary material for: Evaluating an evidence-based curriculum in undergraduate palliative care education: piloting a phase II exploratory trial for a complex intervention
Source: BMC Med Educ. 2013 Jan 4;13:1. doi: 10.1186/1472-6920-13-1 (PMC3546306; doi:10.1186/1472-6920-13-1)
Supplement: Additional file 1 — Manual_UPCE_curriculum_Witten_2012.pdf. A manualised model curriculum in Undergraduate Palliative Care Education at Witten/Herdecke University. PDF-viewer required. [file 1472-6920-13-1-S1.docx]

Undergraduate Palliative Care Education

- A manualised model curriculum in Undergraduate Palliative Care Education at Witten/Herdecke University

Mischa Möller^1^, Martin W. Schnell^1^, Christian. Schulz^2,3^

^1^Institute for Ethics and Communication in Healthcare Systems, School of Medicine, Faculty of Health, Witten/Herdecke University, Alfred-Herrhausen-Straße 50, 58448 Witten, Germany.
[mischa.moeller@uni-wh.de](mailto:mischa.moeller@uni-wh.de); [martin.schnell@uni-wh.de](file:///C:\Dokumente%20und%20Einstellungen\Mischa\Eigene%20Dateien\IEKG%20und%20Intergriertes%20Curriculum\AG%20Lebensende%20-%20Palliative%20Care\BMC_ME_An%20evaluated%20curriculum%20in%20Undergraduate%20Palliative%20Care%20Education\martin.schnell@uni-wh.de)

^2^ Interdisciplinary Centre for Palliative Care, Heinrich-Heine-University Düsseldorf, Moorenstraße 5, 40225 Düsseldorf, Germany.
[christian.schulz@med.uni-duesseldorf.de](mailto:christian.schulz@med.uni-duesseldorf.de)

^3^ Univ Dusseldorf, Medical Faculty, Clinical Institute for Psychosomatic Medicine and Psychotherapy, Moorenstraße 5, D-40225 Dusseldorf, Germany

Figure 1: Medical students during the seminar “Communication with the dying patient“

| **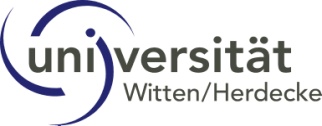** | 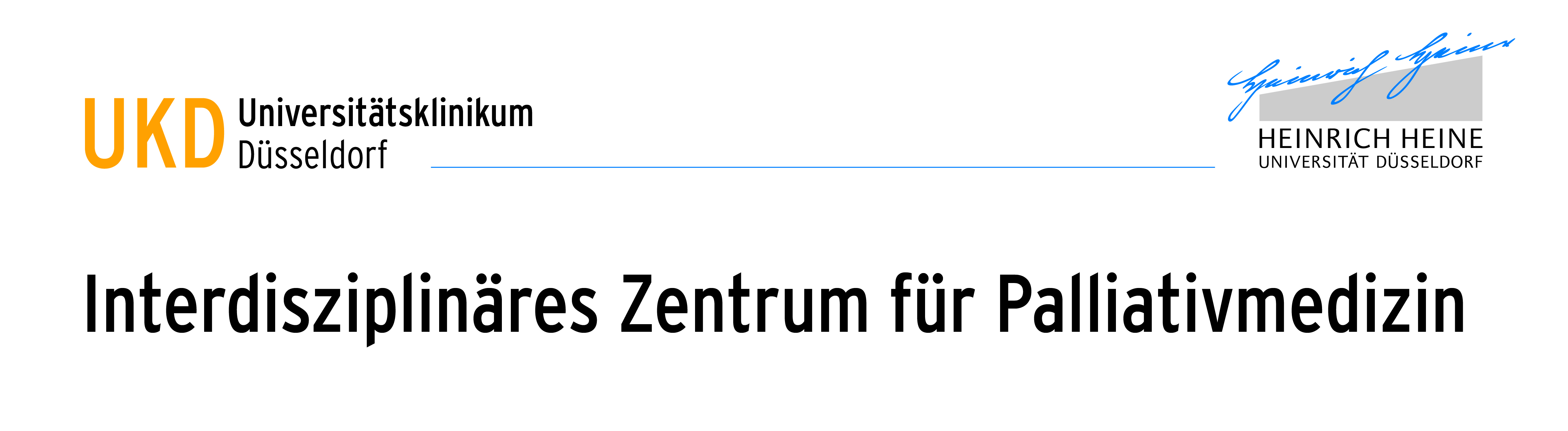 |
| --- | --- |
| **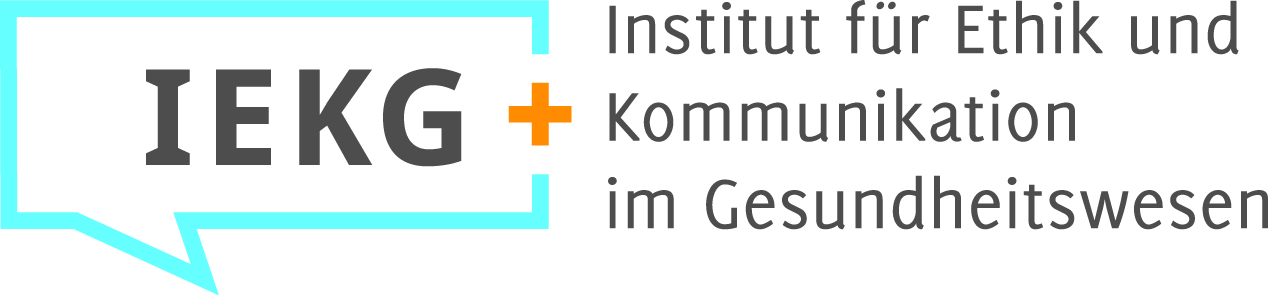** |  |

**Index**

**1. Introduction 3**

**2. Communication and interaction 5**

**2.1 Delivering difficult News**

**2.2 Communication with the dying patient**

**3. Patient 8**

**3.1 Assessment in Palliative Care**

**3.2 Symptom- and Pain management**

**4. Inter-professionalism 10**

**5. System 12**

**5.1 Family-centred medicine**

**5.2 Legal aspects at the end of life**

**5.3 Health economy of death and dying**

**Literature 14**

**Tables 15**

1. **Introduction**

The Undergraduate Palliative Care Education (UPCE) curriculum was first introduced in 2006 at Witten/Herdecke University. Based on a systematic review of literature we developed this manualised curriculum according to Kern’s approach to curriculum development, a six step framework for evidence-based curricular development [1]. Four domains evolved during our investigation summarised in figure 1: (1) communication and interaction, (2) patient assessment and management, (3) inter-professionalism and (4) systemic aspects. The curriculum consists of a total of 31 teaching units (TU=45mins) taught to 4th year medical students during the course of 2 semesters. 10 units were devoted to communication teaching including a longitudinal 1:1 real patient contact module of four months which encircles and links the contents of the curriculum. According to recommendations for teaching communication in medicine preferred methods should be interactive, focussing on group discussion, teamwork, role-play and patient exposure [2]. To get an overview about methods assessing communication in medical professionals please see table 1 at the end of this paper.

Figure 2: Overview of the Undergraduate Palliative Care Education curriculum

Real patient contact as a training, assessment and research method has been used in different palliative care settings [3-5]. The method of direct medical student-patient interaction in communication training is not new. Participation of cancer volunteers in teaching communication skills has been shown to be beneficial and has been demonstrated to have enduring effects on the students [6, 7]. Retrospective analysis of senior student’s perception of adequacy of UPCE found that problem-based-learning, basic science and patient interviewing courses are least effective in affecting competency in Palliative Care [8]. Experimental opportunities through patient experience seminars [4] or clinical rotations [9, 10] have been suggested to provide a more promising impact. A series of publications covers detailed analyses of modules of the curriculum [11]. Qualitative analyses of encounters between dying patients and medical students during real patient contact have been covered by a master thesis at King’s College London and the intervention in interprofessional education of the curriculum has been studied elsewhere [12-14]. More detailed information of the content and scientific basis of the UPCE curriculum can be found in the corresponding literature [German textbook]:

Schnell MW, Schulz C: Basiswissen Palliativmedizin. Springer Verlag , 2011. German.

1. **Communication and interaction**

**2.1 Delivering difficult News**

The communication of Delivering difficult News (DDN) to the patient is a central element of patient care in all disciplines. Good communication is not a trait attribute but can be learned through professional training.

**Setting:** max. 20 students, min. 2 lecturers with expertise in teaching Palliative Care or Psycho-oncology

**Methods:**  interactive seminar, role-play, group discussion, simulated patient- contact

**Learning objectives:** awareness, reflection, concept-building, identifying conflicts, adoption of basic actions

**Table of contents:**

| TU | | **Content** |
| --- | --- | --- |
| 1 | - Welcome - Introduction of feedback-rules - Establishment of a comfortable learning environment - Introduction into the difficulty of *Delivering difficult News* (DDN) | |
| 1 | - Communication-needs of patients and their relatives in Palliative Care - Explaining the concept of patient-centred communication - The SPIKES-model - Talking about prognosis | |
| 1 | - Simulated patient-contact of a situation in DDN - Role-play in small groups - Reflection and feedback | |

**2.2 Communication with the dying patient**

Communication with dying patients introduces diversity into the patient-doctor relationship. Recognition of this reaction may help building a supporting environment for the patient.

**Setting:** max. 20 students, min. 2 lecturers with expertise in teaching Palliative Care or Psycho-oncology, access to dying patients (ECOG 2-3)

**Methods:**  Role-play, reflection in small groups, real patient contact (1:1 interviews), encounter diary

**Learning objectives:** death-awareness, basic understanding of end-of-life diversity, reflection, concept-building of communication models, identifying conflicts, learning and practicing of active listening

**Table of contents:**

| TU | | **Content** |
| --- | --- | --- |
|  | **Part I – Preparing for patient interviews** | |
| 1 | - Welcome and feedback-rules reminder - Exchange of own death-experiences - Ideas about a ”good death“ - Critical reflection of subjective beliefs about dying and death | |
| 1 | - Lecture: *End-of-life Diversity* - Discussion | |
| 2 | - Introduction about the importance of active listening - Emotional competence: the NURSE-model - Shared-decision-making: the OPTION-model - Role-play | |
| 1 | - Existential phenomena at the end-of-life in patients and doctors - Discussion - Optional movie | |
|  | **Part II – Real patient contact module** | |
|  | Students were introduced to dying patients (ECOG 2-3) in a controlled setting under permanent support by physicians and psycho-oncologists. Students are advised to practice active listening and are encouraged to ask questions about death and dying in at least 3 interviews in 4 months. The objective of the interviews is clarified to voluntary participating patients. Students are invited to prepare an encounter diary about content, mood and patient-relationship during the interviews. Diaries will be reviewed by the lecturer for part III without grading. | |
|  | **Part III – Reflection and Feedback** | |
| 1 | - Small group discussion to exchange experiences of the patient interviews - Presentation of didactic examples from the reflective diaries | |
| 1 | - Small group discussion about fear of dying and spirituality - Reflexion and farewell | |

Figure 3: This seminar is split into three consecutive blocks over a period of 4 months.

1. **Patient**

**3.1 Assessment in Palliative Care**

Assessment of patient needs is a basic principle in Palliative Care. This seminar present the most important assessment instruments in that field.

**Setting:** 1 lecturer with professional training in Palliative Care

**Methods:**  lecture, interactive seminar

**Learning objectives:** recognition of the importance of assessment, first experiences with common assessment instruments.

**Table of contents:**

| TU | | **Content** |
| --- | --- | --- |
| 2 | - Welcome and presentation of learning objectives - Types of test-instruments - Quality criteria of test-instruments - Test instruments: Numeric Rating Scale, Visual Analogue Scale, Karnofsky Performance Status, Eastern Cooperative Oncology Group status, Palliative Outcome Scale, HOPE (German: “Hospiz und Palliativ-Erfassung”), Palliative Prognostic Scale, Palliative Prognostic Index | |

**3.2 Symptom- and Pain management**

Once a symptom is identified by the Palliative Care team measures for relief have to be taken. This seminar focuses on prevalent symptoms in Palliative Care and discusses treatment possibilities.

**Setting:** 1-2 lecturers with professional training in Palliative Care

**Methods:**  interactive seminar, role-play, case studies

**Learning objectives:** recognition of typical symptoms in Palliative Care, first experiences

in treatment possibilities

**Table of contents:**

| TU | | **Content** |
| --- | --- | --- |
| 2 | - Welcome and presentation of learning objectives - Feedback-rules reminder - Basics of symptom-management: basics, pharmacological therapy, subcutaneous application, medical infusion pumps - Introduction in typical symptoms in Palliative Care: fatigue, pain, anorexia, nutrition, nausea and vomiting, obstipation, obstruction/ileus, diarrhoea, dyspnoea, tussis, fear, depression, delirium, epilepsy, wounds, itching, thirst, oral care - Symptoms in the final phase of dying | |
| 3 | - Interactive virtual case studies in small groups (ppt presentations of real cases are preferred over paper cases) - Elucidation of therapy options and strategies | |

1. **Inter-professionalism**

**Delivering palliative care to elderly patients**

Delivering Palliative Care to elderly patients is a complex task for the interdisciplinary Palliative Care team. While the number of elderly patients is increasing in many western countries multimorbidity, dementia and frailty complicate care. This seminar was designed to strengthen the cooperation between individuals of different professions to satisfy the palliative care needs of the elderly.

**Setting:** 2 lecturers, one with professional training in Palliative Care and one in Geriatrics, access to nursing students for interprofessional approach

**Methods:**  experimental interprofessional education seminar [13, 14]

**Learning objectives:** learning about the burden of the old, competences of teamwork, interprofessional communication, interprofessional case conference

**Table of contents:**

| TU | | **Content** |
| --- | --- | --- |
| 1 | - Welcome and presentation of learning objectives - Feedback-rules reminder - Introduction to the palliative care needs of elderly patients - Management of multimorbidity - Geriatric assessment | |
| 1 | - The idea of holistic care - Pain-management of the elderly | |
| 2 | Interprofessional core competencies conducted through experimental interprofessional education:   - Respect - Communication - Patient-centred practice - Decision-making - Shared knowledge and skills - Problem solving - Working collaboratively in a team | |

1. **System**

**5.1 Family-centred medicine**

The unit of care in Palliative Care is defined as seriously ill and dying patient including their relatives according to the definition from the World Health Organisation. Family members often are extremely affected by the suffering of their loved ones. This seminar helps the students to understand how the family-system can be supported by the surrounding Palliative Care team.

**Setting:** 1 lecturer with professional training in Palliative Care

**Methods:**  interactive seminar

**Learning objectives:** perception of a systemic perspective on families, understanding the needs of families caring for a seriously ill member

**Table of contents:**

| TU | | **Content** |
| --- | --- | --- |
| 1 | - Welcome and presentation of learning objectives - Feedback-rules reminder - Introduction in Family-centred medicine | |
| 2 | Requirements of professional family-centred medicine:   - Knowledge about systems theory - Communication strategies dealing with families - Self-reflexion of own family structures - Resources - Needs of family members, - Bereavement - Information needs - Privacy | |

**5.2 Legal aspects at the end of life**

Palliative Care has to consider the legislation of the specific country to full-fill its task.

**Setting:** 1 lecturer with professional training in Palliative Care

**Methods:**  interactive seminar, virtualized real case scenarios

**Learning objectives:** to understand the specific legal aspects underlying Palliative Care practice.

**Table of contents:**

| TU | | **Content** |
| --- | --- | --- |
| 2 | - Welcome and presentation of learning objectives - Feedback-rules reminder - Introduction to legal aspects important for Palliative Care: *“Patientenverfügung“* (personal and advanced directive), *“Vorsorgevollmacht“* and *“Betreuungsverfügung“* (health care proxy) | |
| 2 | - Case studies of complex treatment decisions in Palliative Care situations (preferably cases in which something went wrong or ethical discussion could not solve the conflict entirely) | |

**5.3 Health Economy of Death and Dying**

Palliative Care in Germany must be considered in context of the healthcare system. This seminar classifies financial structures of the German healthcare system and connects them with Palliative Care.

**Setting:** 1 lecturer with professional training in Palliative Care

**Methods:**  seminar

**Learning objectives:** Health Economy with specific expertise in the domain of hospice work and palliative care

**Table of contents:**

| TU | | **Content** |
| --- | --- | --- |
| 1 | - Welcome and presentation of learning objectives - Presentation of financial structures of the healthcare system in Germany | |
| 2 | - Introduction of specific funding of Palliative Care in outpatient and hospital care | |

**Literature**

1. Kern DE, Thomas PA, Howard DA: *Curriculum Development for Medical*

*Education - A Six-Step Approach*: John Hopkins University Press; 1998.

2. Kurtz SM, Silverman J, Draper J: *Teaching and learning communication skills in medicine*. 2nd edition. Oxford: Radcliffe; 2005.

3. Ross DD, Keay T, Timmel D, Alexander C, Dignon C, O'Mara A, O'Brien W, 3rd: **Required training in hospice and palliative care at the University of Maryland School of Medicine**. *J Cancer Educ* 1999, **14**(3):132-136.

4. Cowell DD, Farrell C, Campbell NA, Canady BE: **Management of terminal illness: a medical school-hospice partnership model to teach medical students about end-of-life care**. *Acad Psychiatry* 2002, **26**(2):76-81.

5. Block SD, Billings JA: **Learning from the dying**. *N Engl J Med* 2005, **353**(13):1313-1315.

6. Bickel-Swenson D: **End-of-life training in U.S. medical schools: a systematic literature review**. *J Palliat Med* 2007, **10**(1):229-235.

7. Klein S, Tracy D, Kitchener HC, Walker LG: **The effects of the participation of patients with cancer in teaching communication skills to medical undergraduates: a randomised study with follow-up after 2 years**. *Eur J Cancer* 1999, **35**(10):1448-1456.

8. Fraser HC, Kutner JS, Pfeifer MP: **Senior medical students' perceptions of the adequacy of education on end-of-life issues**. *J Palliat Med* 2001, **4**(3):337-343.

9. Ross DD, O'Mara A, Pickens N, Keay T, Timmel D, Alexander C, Hawtin C, O'Brien W, 3rd, Schnaper N: **Hospice and palliative care education in medical school: a module on the role of the physician in end-of-life care**. *J Cancer Educ* 1997, **12**(3):152-156.

10. Porter-Williamson K, von Gunten CF, Garman K, Herbst L, Bluestein HG, Evans W: **Improving knowledge in palliative medicine with a required hospice rotation for third-year medical students**. *Acad Med* 2004, **79**(8):777-782.

11. Schulz C, Möller MF, Schmincke-Blau I, Schnell MW: **Communication with the dying patient – Results of a controlled intervention study on communication skills in undergraduates**. In *European Journal of Palliative Care. Volume 11*. Edited by Nauck F. Vienna: Hayward Medical Communications; 2009:152.

12. Schulz C: **The encounter between dying patients and medical undergraduates during a course in end-of-life communication in the medical curriculum: a qualitative approach to insights into the patient perspective.** King's College, Department of Palliative Care, Rehabilitation, Policy & Rehabilitation; 2010.

13. Just JM, Schnell MW, Bongartz M, Schulz C: **Exploring Effects of Interprofessional Education on Undergraduate Students Behaviour: A Randomized Controlled Trial.** *Journal of Research in Interprofessional Practice and Education* 2010, **1**(3):182-199.

14. Just JM, Schulz C, Bongartz M, Schnell MW: **Palliative care for the elderly--developing a curriculum for nursing and medical students**. *BMC Geriatr* 2010, **10**:66.

15. Epstein RM: **Assessment in medical education**. *N Engl J Med* 2007, **356**(4):387-396.

**Tables**

| **Available methods for evaluation and assessment of communication** | | | | |
| --- | --- | --- | --- | --- |
| **Method** | **Domain** | **Pros** | **Cons** | **Validity/Reliability** |
| **Self-assessment** |  |  |  |  |
| Self-applied questionnaire | Knowledge, skills, attitude, beliefs, emotions, behaviour | Easy to apply, cost-effective, data easily accessible, foster reflection, access to sensitive data through anonymity | Accuracy questionable, over-estimation-effects if no training and feedback, direct practice-conclusions not possible | Most frequently used instrument with numerous validated and reliable instruments (but: do we really measure what we want to measure?) |
| Reflection diary | Attitudes, beliefs, behaviour, emotions, clinical reasoning | Easy to apply, cost-effective, fosters reflection and development of learning plans, generates in-depth data | Time-consuming, strong biases possible through selection, interpretation and defence-reactions | Not applicable |
| Video log (self-recorded video sequences comparable to a video diary) | All domains, especially intra- and interpersonal dynamics | Individual recording timing, in-depth data, mimic and gesture data | Logistically challenging, high costs, time-consuming, very complex qualitative data | No studies identified / not applicable |
| **Patient-assessment** |  |  |  |  |
| Patient questionnaire | Patient satisfaction/ quality of life, rapport building, behaviours, patient priorities, interpersonal communication | Relevant and important source of assessment | Tendency to give global impression rather than analysis, ethical challenges, low discriminatory power | Validated and reliable instruments exist and have been used in various settings^26^ |
| Patient interview | All patient-centred domains | Rich in-depth-data including mimic and gesture (in video), qualitative approach which fosters learning and understanding | Highly time and cost consuming, Ethical concerns (anonymity, vulnerability), not applicable in large groups | Not used in formal assessment |
| **Peer assessment** |  |  |  |  |
| Group discussion | Professionalism, performance, teamwork, interpersonal behaviour, systemic aspects | Credible source, ratings encompass habitual behaviours, realistic feedback setting | Setting must be secure (confidentiality, trust, feedback-rules), time-consuming | correlates with future academic and clinical performance |
| **Supervisor assessment** |  |  |  |  |
| Multiple Choice Questions | Knowledge, problem-solving | Time-efficient, can cover different content areas in little time, allows high output, clear rating scales, can be automated, high discriminatory power | Development of highly valid/reliable questions is a great challenge, especially in topics like communication, cueing-effect possible (right answer only if options are presented) | High reliability, can also reach high validity if properly constructed^27^ |
| Key Feature^28^  (sequential patient case information items are intermitted by questions about clinically relevant decisions) | Knowledge, problem-solving, clinical reasoning | Can cover different content areas in little time, assess problem-solving-abilities, avoids cueing | Time-consuming developmental process | High reliability and validity possible if well constructed^27^ |
| Vignettes/ structured essays  (special form: objective structured video examinations= OSVEs)^29^ | Synthesis and integration competence, clinical reasoning, knowledge application | Realistic case presentation with complex data (especially in OSVEs) | Time-consuming for preparation/development (OSVEs), time-consuming for grading | Interrater reliability is a problem, needs many pre-tests, high validity if well constructed cases^27^ |
| Real patient contact (RPC)^30,31^ as structured direct observation (mini-clinical-evaluation exercise [mini-CEX]^35^ or video review^36^) | Skills, interpersonal communication | Real patients, real cases, specifically valuable feedback, has enduring effect on students^32^ | Complex and time-consuming, ethical issues, might observe selective instead of habitual behaviour (performance when not observed) | High reliability^33,34^ |
| **Clinical simulations** |  |  |  |  |
| Simulated/Standardised patient contact (SPC)^37^ | Skills, interpersonal behaviour, attitude, communication skills | Can be very realistic if well designed and prepared, structured rating, transparent rating criteria, can be recorded and debriefed | Time-consuming, cost-intensive development, preparation and realisation, artificial setting, selective behaviour (see above) | Reliable and consistent, high validity if well constructed case^38^ |
| Incognito SPC^39,40^ | Habitual behaviour in actual practice | Realistic, accurate, combines advantages of simulation and realistic scenarios | Expensive and logistically very demanding, ethical concerns (concerning both stimulant and participant) | No data |
| Objective structured clinical examination (OSCE)^41^ | Knowledge, skills, interpersonal communication | Perceived positively by students and teachers, high interactivity, can be very realistic | Very time- and cost-consuming, scores differentiate between examinees with different clinical levels of expertise, discrepancy between OSCE scores and communication skills as perceived by patients^42^ | High reliability, high validity^43,44^ |
| Complete simulation settings (e.g. simulation hospitals) | Knowledge, skills, teamwork, systemic aspects | Very realistic setting, complex scenarios can be simulated, integrative and meta-competencies can be tested | Most expensive and complex method | No data |

Table 1: Available methods for evaluation and assessment of communication with dying patients (adapted from [15])
