# Supplementary material for: Evaluating an evidence-based curriculum in undergraduate palliative care education: piloting a phase II exploratory trial for a complex intervention
Source: BMC Med Educ. 2013 Jan 4;13:1. doi: 10.1186/1472-6920-13-1 (PMC3546306; doi:10.1186/1472-6920-13-1)
Supplement: Additional file 2 — Medical_interest_of_participants.pdf. Prior to the intervention the medical interest of participants was evaluated. PDF-viewer required. [file 1472-6920-13-1-S2.doc]

## Additional file 2 –

## Medical interest of participants

|  | IG (n=15) | CG (n=22) | total (n=37) |
| --- | --- | --- | --- |
| Family medicine | 2 | 0 | 2 |
| Anaesthesia | 0 | 2 | 2 |
| Surgery | 2 | 3 | 5 |
| Research | 0 | 2 | 2 |
| Gynaecology | 0 | 1 | 1 |
| Haematology | 0 | 1 | 1 |
| Internal medicine | 1 | 3 | 4 |
| Cardiology | 0 | 1 | 1 |
| Neurosurgery | 1 | 1 | 2 |
| Neurology | 1 | 1 | 2 |
| Oncology | 0 | 3 | 3 |
| Paediatrics | 5 | 3 | 8 |
| Palliative care | 2 | 2 | 4 |
| Psychiatry | 1 | 4 | 5 |
| Public Health | 1 | 0 | 1 |
| Tropical medicine | 1 | 0 | 1 |
| Trauma surgery | 3 | 3 | 6 |
| Urology | 1 | 2 | 3 |
| “don’t know yet” | 1 | 0 | 1 |
