# Supplementary material for: Evaluating an evidence-based curriculum in undergraduate palliative care education: piloting a phase II exploratory trial for a complex intervention
Source: BMC Med Educ. 2013 Jan 4;13:1. doi: 10.1186/1472-6920-13-1 (PMC3546306; doi:10.1186/1472-6920-13-1)
Supplement: Additional file 3 — Post-interventional_intergroup-comparison_T2.pdf. Surplus statistical results, confirming the main results. PDF-viewer required. [file 1472-6920-13-1-S3.doc]

## Additional file 3 –

## Post-interventional intergroup-comparison at T2

Post-interventional comparison of IG and CG at T2 shows statistical significant results for constructs (2), (3) and (4) in accordance with the previous results in table 7. Data of (1) is not presented because that construct has proved to be unreliable.

| Construct (No.) | Groups | T1 M ± SD | *t* | *Df* | *p*-value | MD | 95% CI |
| --- | --- | --- | --- | --- | --- | --- | --- |
| (2) | IG  CG | 37.3 ± 6.65  24.19 ± 7.53 | 4.65 | 21.08 | <.001 * | 13.11 | 7.11, 19.11 |
| (3) | IG  CG | 23.6 ± 5.58  13.69 ± 6.12 | 4.24 | 20.62 | <.001 * | 9.91 | 5.05, 14.78 |
| (4) | IG  CG | 13.2 ± 2.7  8.75 ± 2.89 | 3.98 | 20.25 | .001 * | 4.45 | 2.12, 6.78 |
| (5.1) | IG  CG | 20.0 ± 4.19  23.75 ±6.28 | -1.83 | 23.83 | .08 | -3.75 | -7.99, .49 |
| (5.2) | IG  CG | 14.0 ± 4.32  15.63 ± 6.12 | -.79 | 23.53 | .44 | -1.63 | -5.86, 2.61 |
| (5.3) | IG  CG | 15.9 ± 3.96  17.88 ± 5.11 | -1.1 | 22.73 | .28 | -1.98 | -5.68, 1.73 |
| (5.4) | IG  CG | 21.8 ± 4.71  20.25 ± 5.64 | .76 | 21.84 | .46 | 1.55 | -2.7, 5.8 |

Legend: (2) Willingness to accompany dying patients; (3) Self-estimation of competence in communication with dying patients and their relatives; (4) self-estimation of knowledge and skills in PC; (5.1) Attitude towards death of self; (5.2) Attitude towards dying of self; (5.3) Attitude towards death of others; (5.4) Attitude towards dying of others. M=mean; SD=standard deviation; MD=mean difference; * significant.
