# Supplementary material for: Evaluating an evidence-based curriculum in undergraduate palliative care education: piloting a phase II exploratory trial for a complex intervention
Source: BMC Med Educ. 2013 Jan 4;13:1. doi: 10.1186/1472-6920-13-1 (PMC3546306; doi:10.1186/1472-6920-13-1)
Supplement: Additional file 4 — Changes_in_emotional_involvement.pdf. Emotional changes measured by the modified Differential Emotions Scale of the participants while attending the seminar “Communication with the dying patient”. PDF-viewer required. [file 1472-6920-13-1-S4.doc]

## Additional file 4 –

## Changes in emotional involvement

As secondary outcome we measured changes in emotional involvement of the participants while attending the seminar “Communication with the dying patient” which fosters death-awareness. Therefore the IG was assessed by a modified version of Izard’s Differential Emotions Scale (mDES) pre and post {Boyle, 1984 #61}. The mentioned mDES is an instrument to assess emotional involvement introduced 1974 by C.E. Izard in an unpublished manuscript at Vanderbilt University (USA). The mDes was improved in 2006 by D. Renaud and D. Unz at the University of Saarland (Germany) to measure constructs of positive emotions. The mDES assesses 15 emotional scales, each consisting of three items, giving a total of 45 items designed as semantic differential in five-point Likert scales. The mDES was only used pre and post the seminar “Communication with the dying patient” in the IG. Data obtained from the mDES of the IG was tested by Wilcoxon test with a significance margin of *p*<.05.

## Changes in emotional involvement while attending the seminar “Communication with the dying patient” measured by mDES (n=17)

| Emotional scales | pre  M±SD | post  M±SD | *p*-value |
| --- | --- | --- | --- |
| joy | 7.29 ±2.2 | 7.29 ± 2.34 | .94 |
| happiness | 9.82 ± 2.13 | 8.88 ± 2.8 | .19 |
| satisfaction | 10.24 ± 2.05 | 10.0 ± 2.67 | .64 |
| sympathy | 8.76 ± 2.86 | 8.59 ± 3.2 | .53 |
| fascination | 7.53 ± 2.98 | 9.35 ± 2.37 | .046 * |
| affection | 7.59 ± 2.92 | 9.53 ± 2.48 | .034 * |
| Interest | 11.76 ± 2.05 | 9.18 ± 2.35 | .004 * |
| surprise | 6.47 ± 2.79 | 7.65 ± 3.02 | .24 |
| grief | 5.24 ± 2.44 | 6.41 ± 2.53 | .033* |
| anger | 4.47 ± 1.775 | 4.24 ± 1.3 | .60 |
| aversion | 3.71 ± 1.21 | 3.35 ± .79 | .16 |
| contempt | 3.35 ± 1.06 | 3.12 ± .6 | .38 |
| fear | 5.76 ± 2.19 | 5.41 ± 1.5 | .39 |
| boredom | 4.18 ± 1.19 | 3.82 ± 1.19 | .4 |
| shame | 5.82 ± 1.94 | 5.0 ± 1.37 | .036* |
| guilt | 4.47 ± 1.62 | 3.71 ± .77 | .098 |

Legend: n=17; M=mean; SD=standard deviation; * significant.

The participation of the seminar “Communication with the dying patient” fostered emotional involvement in fascination, affection and grief, while measures of interest and shame were reduced. Significant changes in the following emotional dimensions were measured: fascination rose from 7.53 (SD 2.98) to 9.35 (SD 2.37), *p* = .05. Affect rose from 7.59 (SD 2.92) to 9.53 (SD 2.48), *p* = .03. Grief rose from 5.24 (SD 2.44) to 6.41 (SD 2.53), *p* = .03. Interest sank from 11.77 (SD 2.05) to 9.18 (SD 2.35), *p* < .01. Shame sank from 5.82 (SD 1.94) to 5.00 (SD 1.37), *p* = .04. Further information is listed in the table.
